# Multiple sclerosis clinical forms classification with graph convolutional networks based on brain morphological connectivity

**DOI:** 10.3389/fnins.2023.1268860

**Published:** 2024-01-18

**Authors:** Enyi Chen, Berardino Barile, Françoise Durand-Dubief, Thomas Grenier, Dominique Sappey-Marinier

**Affiliations:** ^1^CREATIS, CNRS UMR 5220, INSERM U1294, Université de Lyon, Université Claude Bernard-Lyon 1, INSA Lyon, Lyon, France; ^2^Service de Sclérose en Plaques, des Pathologies de la Myéline et Neuro-Inflammation, Groupement Hospitalier Est, Hôpital Neurologique, Bron, France; ^3^CERMEP - Imagerie du Vivant, Université de Lyon, Bron, France

**Keywords:** multiple sclerosis, graph convolutional network, CNN, classification, brain morphological connectivity, gray matter thickness

## Abstract

Multiple Sclerosis (MS) is an autoimmune disease that combines chronic inflammatory and neurodegenerative processes underlying different clinical forms of evolution, such as relapsing-remitting, secondary progressive, or primary progressive MS. This identification is usually performed by clinical evaluation at the diagnosis or during the course of the disease for the secondary progressive phase. In parallel, magnetic resonance imaging (MRI) analysis is a mandatory diagnostic complement. Identifying the clinical form from MR images is therefore a helpful and challenging task. Here, we propose a new approach for the automatic classification of MS forms based on conventional MRI (i.e., T1-weighted images) that are commonly used in clinical context. For this purpose, we investigated the morphological connectome features using graph based convolutional neural network. Our results obtained from the longitudinal study of 91 MS patients highlight the performance (F1-score) of this approach that is better than state-of-the-art as 3D convolutional neural networks. These results open the way for clinical applications such as disability correlation only using T1-weighted images.

## 1 Introduction

Multiple sclerosis (MS) is a chronic autoimmune inflammatory and demyelinating disease of the central nervous system. While its etiology is still unknown (Polman et al., 2011), MS is the first cause of non-traumatic neurological disability in young adults, affecting about 2.8 million people worldwide (Goodin, 2014). Often starting with a preliminary clinical isolated syndrome (CIS) involving a large heterogeneity of clinical symptoms such as weak limbs, blurred vision, dizziness, fatigue, or tingling sensations, the disease may evolve along two main clinical courses. In 85% of patients, the disease starts as a relapsing-remitting course (RRMS, noted RR), with the occurrence of relapses. These RRMS patients can evolve over time into a non-systematic secondary-progressive course (SPMS, noted SP). In the 15% remaining patients, the disease evolves as primary-progressive MS (PPMS, noted PP) which corresponds to a steadily worsening of symptoms over time without any relapses (Lublin et al., 2014). The current McDonald diagnostic criteria for MS combine clinical assessment, imaging, and laboratory findings (Thompson et al., 2018). Despite such clinical classification, the status and the evolution of each patient could be very different from one to another, leading more and more to individual therapeutic approaches. Thus, to propose personalized medical care and therapy, the neurologist needs to better predict the disease evolution based on early clinical, biological, and imaging markers available from disease onset.

Magnetic Resonance Imaging (MRI) is the most effective tool for the diagnosis of MS and for monitoring the disease modifying treatment. Conventional MRI provides T1-weighted (T1w), T2-weighted (T2w) and FLAIR images allowing the detection and follow-up of white matter (WM) lesions for clinical care (Mure et al., 2016). These conventional sequences allow the quantification of whole brain, WM or gray matter (GM) atrophy using dedicated software. More advanced MRI sequences such as diffusion-weighted imaging (DWI) and diffusion tensor imaging (DTI) have been developed to provide more sensitive markers of the inflammation processes occurring in WM and leading to T1- and T2-lesions. Several metrics of DTI such as the fractional anisotropy and the mean diffusion enable the detection of micro-architectural alterations in WM lesions as well as in normal-appearing WM (Jutten et al., 2019).

More recently, graph theory methods have been used to model brain network organization (Rubinov and Sporns, 2010; Guo et al., 2017). These graph models consist of nodes, based on the parcellation of brain GM regions, and edges, determined by the underlying links between the network nodes. In brain structural connectivity, these links are defined by the extraction of WM fibers using DTI tractography (Hagmann et al., 2007). Previously, Kocevar et al. (2016) have demonstrated an interest of such approaches for the classification of MS clinical profiles using Machine Learning (ML) methods, while Marzullo et al. (2019) improved the classification performance by a first approach using a Deep Learning (DL) model.

However, DTI data used for structural connectivity modeling require long acquisition time and complex processing techniques, which limits its applicability in clinical practice. Nevertheless, brain connectivity can also be obtained from conventional MRI by measuring morphological metrics of the GM on T1w images (Raamana and Strother, 2018). Indeed, several imaging investigations have shown that GM atrophy is present early in MS (Durand-Dubief et al., 2012; Eshaghi et al., 2018). Narayana et al. (2013) has found significant cortical thinning in RRMS patients compared to healthy subjects. Hence, the GM degeneration used in brain morphological connectivity models could provide a sensitive marker of the disease evolution. In such graphs, nodes represent GM areas obtained from the GM tissue parcellation, while edges represent a degree of (dis-)similarity between nodes features like GM thickness or curvature (MacDonald et al., 2000). Such approach has been recently used in Alzheimer's Disease (AD), showing that GM network measures predicted hippocampal atrophy rates in preclinical AD, in contrast to other AD biomarkers (Dicks et al., 2020). Also, Mahjoub et al. (2018) proposed to use morphological connectivity to discriminate late mild cognitive impairment from AD patients. Several studies of GM morphological network were used in Autism Spectrum Disorder (ASD) patients. Kong et al. (2019) proposed an auto-encoder-based deep neural network to identify ASD patients from typical controls, while Corps and Rekik (2019) used morphological networks to estimate the ASD patients' age and deduce the age-related cortical regions. In MS, Muthuraman et al. (2016) analyzed morphological GM thickness networks to classify CIS and RRMS patients using the Support Vector Machine model, obtaining a good level of accuracy. Meanwhile, several studies used graph metrics of GM networks to characterize MS patients. Hawkins et al. (2020) found reduced global efficiency and a more random network in RRMS subjects with cognitive impairment. Likewise, lower node degree and connectivity density were found by Rimkus et al. (2019) in MS patients with cognitive impairment. Rocca et al. (2021) combined functional connectivity and GM network to predict clinical worsening in MS, confirming that GM atrophy is an important predictor for the conversion from RRMS to SPMS. By using the source-based morphometry approach to decompose the cortical thickness map into different patterns, Steenwijk et al. (2016) have further shown that several anatomical patterns are strongly associated with clinical dysfunction in MS patients. Meanwhile, several studies also addressed the problem of age/gender and cortical thickness correlation, and removed their effects before further analysis. Eshaghi et al. (2016) fitted the linear regression between age and GM measurements and took only the residual part to classify MS cohort from neuromyelitis optical patients. Given the graph nature of brain connectivity, the use of graph neural network (GNN) to process such data is an evitable path. GNN allows us to deal with the heterogeneity of input data by capturing the message passing across nodes (Bronstein et al., 2021). More specifically, graph convolutional network (GCN), a reimplementation of convolution concept on GNN, is now ubiquitous in solving problems on non-euclidean data.

In the meantime, the application of convolutional neural network (CNN) has proven its strong ability in computer vision, especially in the biomedical image processing field. Leclerc et al. (2019) has successfully delineated cardiac structure on ultrasound images through an encoder-decoder-based model. 3D-CNN, a particular type of CNN, has been widely used in medical context since a huge amount of medical images were acquired and reconstructed in 3 dimensions. Various studies have focused on disease detection from anatomical neuroimaging (Wargnier-Dauchelle et al., 2023). Huang et al. (2019) have built a VGG-like CNN to adapt 3D image challenge for the purpose of Alzheimer's Disease (AD) classification using both T1w-MRI and FDG-PET modalities for a better outcome. Folego et al. (2020) have adapted LeNet, VGGNet, GoogLeNet, and ResNet in 3D domain to the aim of AD detection. Flaus et al. (2022) has proposed a 3D sequential ResNet to enhance PET images for better visualization of brain lesions. A transparent CNN framework proposed by Eitel et al. (2019) has revealed the decision process of CNN in the diagnosis of MS and pointed out more disease-relevant features in MR images. Optic nerve lesions, one of the first manifestations of MS, can be detected by the 3D-CNN model designed by Marti-Juan et al. (2022).

In this study, we proposed to use GCN for the classification of MS clinical forms based only on the measurement of GM morphological feature (thickness) obtained from T1w-MRI. The impacts of different methodological parameters such as the spatial resolution of the GM parcellation atlases and the level of different graph thresholds were compared. Finally, in order to demonstrate the interest of GCN for MS clinical forms classification, we compared the GCN with a classic 3D-CNN approach.

## 2 Materials and methods

Our method was divided into three steps: (i) cortical feature extraction using FreeSurfer (Fischl, 2012); (ii) generation of brain morphological graphs using distance computation and threshold; and (iii) clinical forms classification using GCN.

### 2.1 MRI acquisition and data

The MS patient group (AMSEP) consists of 42 RR, 28 SP, and 21 PP participants included in a longitudinal MRI study. CIS patients (*n* = 12) were included in the RR patient group, in accordance with our clinical expert. Patients (*n* = 3) with change in clinical forms have been removed from the MS group. The patients underwent MR scans on a 1.5T Siemens Sonata system using an 8-channel head-coil at the Lyon CERMEP imaging platform, including a sagittal millimetric 3D-T1 MPRAGE (magnetization prepared rapid gradient echo-MPRAGE) sequence [(TR/TE/TI) = 1970/3.93/1100 ms, flip angle = 15°, field of view (FOV) = 256 × 256 mm, slice thickness = 1 mm, voxel size = 1 × 1 × 1 mm]. Table 1 provides information on the clinical data in further detail. During the first 3 years, MRI exams were performed every 6 months, and every year during the following years. These make up a MS patient dataset of 660 scans in total as detailed in Table 1. A healthy control (HC) group of 21 subjects following the AMSEP protocol was included in this study.

**Table 1:** MS cohort description of 660 scans including relapsing-remitting (RRMS), primary-progressive (PPMS), and secondary-progressive (SPMS) patients.

	**RRMS**	**PPMS**	**SPMS**
Number of patients (F/M)	42 (30/12)	21 (12/9)	28 (11/17)
Number of scans	299	143	218
Mean age at disease onset	28.5	35.0	27.6
Mean age at each scan (range)	35.4 (20.5–53.1)	43.0 (27.8–51.6)	42.9 (28.9–52.2)
Mean disease duration at first scan	4.9	5.6	13.4
Mean disease duration at each scan	7.3	7.5	15.1
EDSS median (range)	2 (0–5.5)	4 (2–7.5)	5.5 (3–8.5)

Another HC group of 314 scans from the IXI dataset (http://brain-development.org/ixi-dataset/) was introduced for the training process (noted IXI). These healthy subjects underwent MR scans on a 1.5T Philips Gyroscan Intera system using a T1w sequence (TR/TE = 9813/4603 ms, flip angle = 8°, 192 phase encoding steps, reconstruction diameter = 240 mm). These make up a HC dataset of 335 scans in total as detailed in Table 2.

**Table 2:** Healthy controls cohort description of 335 T1-weighted MRI including 21 healthy controls (HC-AMSEP) acquired with the same protocol as MS cohort and 314 healthy controls (HC-IXI) obtained from the open-access IXI dataset.

**HC**	**HC-AMSEP**	**HC-IXI**
Number of subject (F/M)	21 (14/7)	314 (175/139)
Number of scans	21	314
Mean age at scan	42.9 (21.6–56.5)	50.8 (20.1–86.2)

### 2.2 Classification using graph-based convolutional network

**Figure 1:**
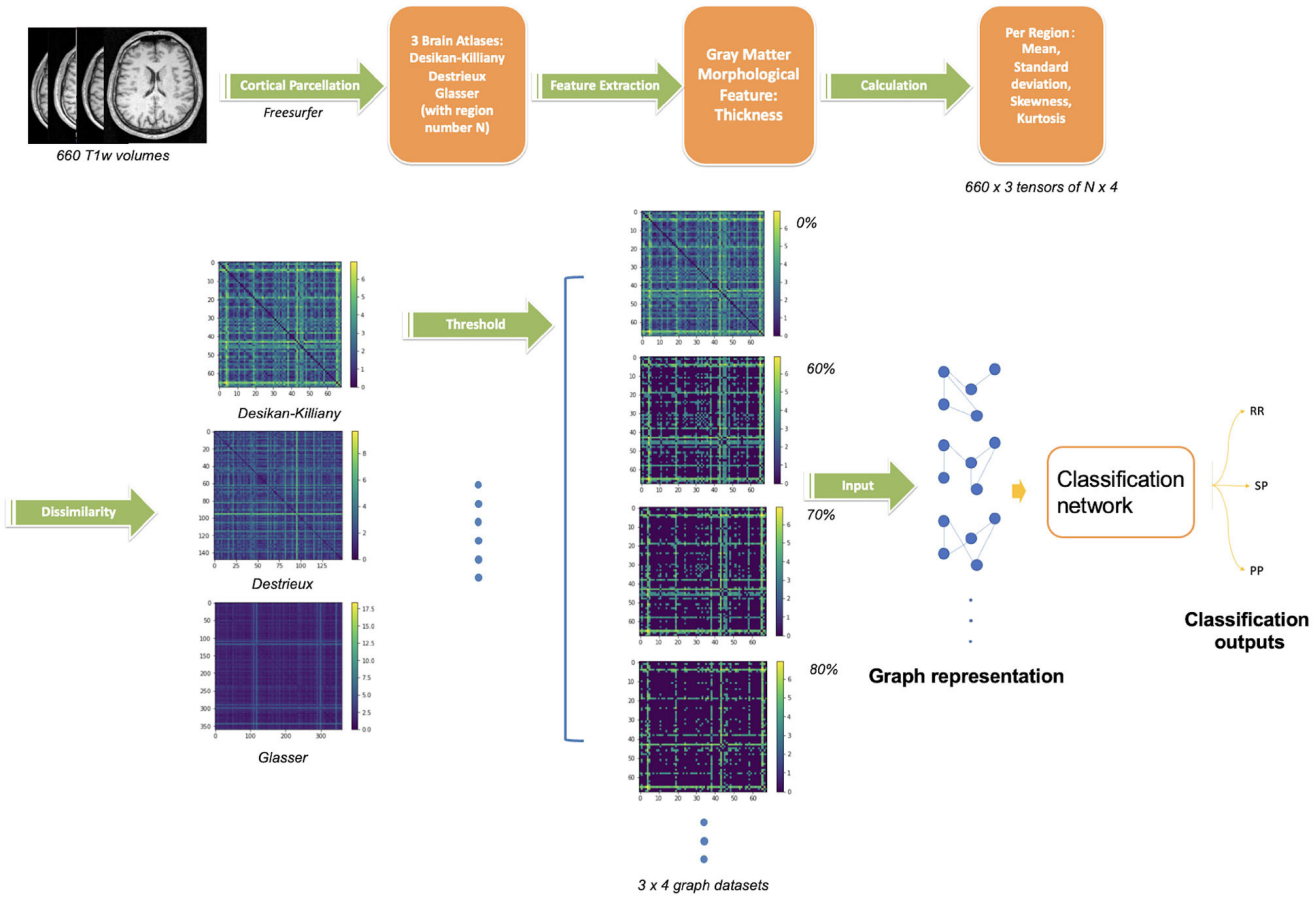
Proposed pipeline for GCN classification. The upper steps illustrate the cortical gray matter regions segmentation from T1w-MRI and parcellation using three atlases, the region feature extraction (thickness) and its vector values. The bottom steps describe the graph construction followed by the GCN classification network. Four threshold levels are applied on graphs (0, 60, 70, 80%), leading to four graphs per atlas. In summary, 12 networks are trained separately (3 atlases, 4 threshold levels) on 660 scans.

As we explore the ability of cortical anatomical changes to identify MS forms, we extract features related to the shape of cortical regions. With such features, we then build a graph reflecting shape similarities between cortical regions and use the graph matrix to train the GCN. The full pipeline of the proposed network is shown in Figure 1.

#### 2.2.1 Feature extraction

In order to obtain features of cortical regions, the brain GM was first segmented (Figure 1), the cortical surface was parcellated into *N* regions using a dedicated brain atlas. Morphological features of each region can thus be calculated and represented as a vector of values.

Automatic segmentation of GM and cortical surface reconstruction were performed on all T1w-MRI using FreeSurfer v6.0.0 image analysis suite (Fischl, 2012), a neuroimaging toolkit for human brain analysis. This includes 31 preprocessing steps such as motion correction, intensity normalization, skull stripping and non-linear registration. All FreeSurfer processing steps were done on the Virtual Imaging Platform (Glatard et al., 2013), the 1,001 images were processed simultaneously and it took 6 h per image on average. The input T1w-MRI brain was resampled onto an average brain (fsaverage) generated from 40 subjects using the Buckner dataset. The Buckner dataset is a subset of a large structural dataset created by the Buckner Lab, it was specifically selected for the intermediate processing step of FreeSurfer. The obtained cortical surface consists of a mesh with 163842 vertices. All outputs were smoothed at full-width/half-max (FWHM) value of 10 mm.

**Figure 2:**
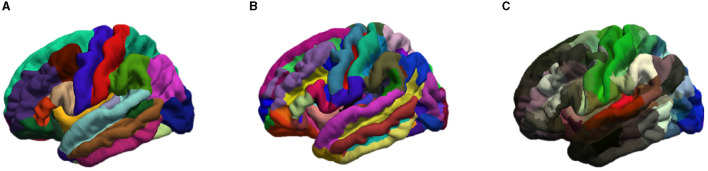
Representation of the cortical parcellation of the three atlases: **(A)** Desikan-Killiany; **(B)** Destrieux; **(C)** Glasser.

These smoothed outputs are then parcellated. In order to study the impact of the number of cortical regions *N*, three different atlases were used for brain parcellation and graph generation, namely the Desikan-Killiany (Desikan et al., 2006) with *N* = 68 regions, Destrieux (Destrieux et al., 2010) with *N* = 148 regions and Glasser (Glasser et al., 2016) with *N* = 360 regions. The cortex parcellation of the average template brain is demonstrated in Figure 2.

More specifically, a region number *i* (with *i* = 1…*N*) was assigned to each vertex according to the atlas chosen by registering the patient's brain mesh to the template brain. As mainly used in brain connectivity studies (reference), the cortical thickness was chosen as the morphological feature and calculated for each region.

Since each region feature is a vector of thousands of elements on average, we summarize the distribution of the thickness values within one region *i* by a vector **x***i* ∈ ℝ^4^ containing the mean value, the standard deviation, the skewness, and the kurtosis: **x***i* = (μ_*i*_, σ_*i*_, γ_*i*_, *k*_*i*_). We called the feature matrix *X* ∈ ℝ^*N*×4^ the combination of the *N* vectors **x***i*.

#### 2.2.2 Age and gender normalization

Since women and men have different cortical atrophy manifestations with age (Narayana et al., 2013), we proposed two methods to normalize **x***i*: a proportional normalization and a residual normalization. For the proportional normalization, we first calculated the average cortical thickness of the whole brain of all MS patients and healthy subjects from the IXI dataset. Then, we performed a linear regression between age and cortical thickness as:


$$\begin{array}{l} Cth=a*age+b \end{array}$$

where *Cth* is the average cortical thickness of one person. Two different sets of coefficients (*a*_*f*_, *b*_*f*_) and (*a*_*m*_, *b*_*m*_) were calculated for healthy women and men respectively. If the slope represents the normal aging effect, we applied this slope to the MS patients group to correct the effect of age and sex. All MS patients' measurements were brought to the age of 20. Thus, the corrected thickness *Cth*_20_ of a patient can be expressed as:


$$\begin{array}{l} Cth_{20}=a*20+b^{\prime }=a*20+Cth-a*age \end{array}$$

Therefore, the adjusted feature vector **x***i*′ of each region with proportional correction with coefficient $\alpha =\frac{Cth_{20}}{Cth}$ can be represented as: $\text{x}i^{\prime }=\left(\alpha \mu_{i},\alpha \sigma_{i},\gamma_{i},k_{i}\right)$. The modified vectors were then used to calculate the new proportional normalized graphs following the same procedure as described above.

Inspired by the work of Eshaghi et al. (2016), we also proposed to adjust each cortical region for the effect of age and gender. For every brain region *i* of the healthy cohort, we fitted a linear regression where age was the regressor and the four attributes of the region were dependent variables. Therefore, for the four values of the feature vector, we have:


$$\begin{array}{l} \mu_{i}=a_{i}^{\left(\mu \right)}*age+b_{i}^{\left(\mu \right)} \end{array}$$

$$\begin{array}{l} \sigma_{i}=a_{i}^{\left(\sigma \right)}*age+b_{i}^{\left(\sigma \right)} \end{array}$$

$$\begin{array}{l} \gamma^{\left(i\right)}=a_{i}^{\left(\gamma \right)}*age+b_{i}^{\left(\gamma \right)} \end{array}$$

$$\begin{array}{l} k_{i}=a_{i}^{\left(k\right)}*age+b_{i}^{\left(k\right)} \end{array}$$

We then estimated the residual of each variable that was inexplicable by the healthy linear regression model: $r_{i}^{\left(\mu \right)}={\hat{\mu }}_{i}-\mu_{i}=a_{i}^{\left(\mu \right)}*age+b_{i}^{\left(\mu \right)}-\mu_{i}$ for example in the case of average cortical thickness measure. The residual feature vector of one region became: $\text{r}i=\left(r_{i}^{\left(\mu \right)},r_{i}^{\left(\sigma \right)},r_{i}^{\left(\gamma \right)},r_{i}^{\left(k\right)}\right)$. The residual vectors were also used to calculate the residual graphs that were further used in the GCN classification. Notice that these regressions are performed for both males and females separately.

#### 2.2.3 Graph generation

A graph *G* is a mathematical representation of a complex system and is defined by a collection of nodes *V* and edges *E* between pairs of nodes with the possibility to assign a weighted value *w* for each edge:


$$\begin{array}{l} G=\left(V,E,w\right) \end{array}$$

Therefore, a brain can be described as a graph, with each brain region being represented by a node **x***i*, or **x***i*′ and **r***i* in case of normalization. Here, we associate four attributes (mean value μ, standard deviation σ, skewness γ, and kurtosis *k*) to each node. The graph representation of brain morphological connectivity was defined as the dissimilarity across brain regions. We propose to compare two distances to calculate the region-wise connections. The first one is the Mahalanobis distance *d*_*M*_:


$$\begin{array}{l} d_{M}\left(\text{x}i,\text{x}j\right)={\left({\left(\text{x}i-\text{x}j\right)}^{T}S^{-1}\left(\text{x}i-\text{x}j\right)\right)}^{1/2} \end{array}$$

with *S* the covariance matrix of samples **x***i* and **x***j*.

The second studied distance is the Taxicab (or Manhattan) distance *d*_*T*_:


$$\begin{array}{l} d_{T}\left(\text{x}i,\text{x}j\right)=\overset{4}{\underset{k=1}{\sum }}|x_{i}^{k}-x_{j}^{k}| \end{array}$$

where $x_{i}^{k}$ is the *k*th dimension of the vector **x***i*.

The adjacent matrix *A* ∈ ℝ^*N*×*N*^ is computed for all distances between **x***i* and **x***j*: *A*(*i, j*)_*X*_ = *d*(**x***i*, **x***j*).

Using both *X* and *A*, we generate weighted and undirected graphs. The edge weights are given by the adjacent matrix.

Thresholds were used to counteract the impact of the redundant information given by the brain adjacent matrix. A fixed rejection quantile τ is used as a threshold value to remove the lowest distances and thus maintains the same graph density for each subject.

For graph availability, the reader can refer to Section 5.

#### 2.2.4 GCN classification

Graph convolutional networks were used as they exploit input data through graph structure. As a dimension reduction tool, graph representation can largely reduce input data size from 12 MB to 130 KB on average in our case. Intuitively speaking, brain network topology is an alternative method of image analysis. Sporns (2018) have confirmed the importance of graph theory for the understanding of brain structure. Based on our previous results using brain structural graph analysis (Marzullo et al., 2019), we explore a new approach using brain morphological graph.

For the graph *G* = (*V, E, w*), the algorithm takes the adjacent matrix *A* and the associated node features matrix *X* as input. The layer-wise propagation rule is defined as follows (Kipf and Welling, 2017):


$$\begin{array}{l} H^{\left(l+1\right)}=\sigma \left({\tilde{D}}^{-\frac{1}{2}}Ã{\tilde{D}}^{-\frac{1}{2}}H^{\left(l\right)}W^{\left(l\right)}\right) \end{array}$$

Where Ã is the sum of *A* with the identity matrix *I*, $\tilde{D}$ is the corresponding diagonal degree matrix and the adjacent matrix is normalized by the step ${\tilde{D}}^{-\frac{1}{2}}Ã{\tilde{D}}^{-\frac{1}{2}}$. *W*^*l*^ represents the trainable weight over each layer. The RELU activation function σ(*x*) = *max*(0, *x*) is chosen for σ.

#### 2.2.5 GCN architecture

**Figure 3:**
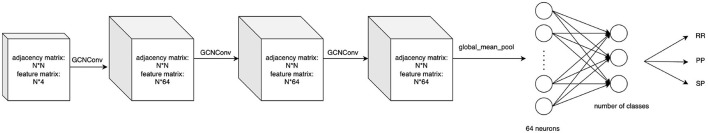
The overall structure of the proposed graph-based convolutional network. *N* is the number of regions according to the atlas chosen. Four represents the four elements of the feature vector per region. Input of the network consists of one adjacency matrix (N*N) and one feature matrix (N*4) per patient. The network starts with three graph convolutional layers of 64 filters each, then gathered into a vector using a global mean pooling. Two fully connected layers are used to obtain the classification into three classes (RR, PP, SP).

The proposed GCN classification model was composed of 3 GCN layers followed by a global mean pool layer with a dropout rate of 0.3 to prevent overfitting. The proposed structure is shown in Figure 3. This led to 8835 trainable parameters.

### 2.3 Classification using 3D convolutional neural network

To validate our GCN against classically used CNN architectures, we implemented a 3D-CNN architecture using a similar architecture by replacing graph convolutional layers with classical convolutional layers. The output of a filter of a 3D convolutional layer with kernel W of size (*f*_*h*_*xf*_*w*_*xf*_*d*_*xf*_*c*_) can be expressed as follows:


$$\begin{array}{l} z_{i,j,k}=b+\overset{f_{h}-1}{\underset{p=0}{\sum }}\overset{f_{w}-1}{\underset{q=0}{\sum }}\overset{f_{d}-1}{\underset{r=0}{\sum }}\overset{f_{c}-1}{\underset{c=0}{\sum }}x_{i^{\prime },j^{\prime },k^{\prime },c}.{\text{W}}_{p,q,r,c} \end{array}$$

with


$$\begin{array}{l} i^{\prime }=i+p-\left\lfloor f_{h}/2\right\rfloor and\;j^{\prime }=j+q-\left\lfloor f_{w}/2\right\rfloor \;and\\ k^{\prime }=k+r-\left\lfloor f_{d}/2\right\rfloor \end{array}$$

Therefore, a 3D-CNN model was constituted of three 3D convolutional layer sets, including a 3D convolutional layer (kernel of 3 × 3 × 3), followed by a max pooling layer (subsampling spatial support by 2 × 2 × 2) and then a batch normalization layer. The tensor is then flattened and used as input of two consecutive fully connected layers of 128 and 2 neurons, respectively. These made up of 22,548,122 trainable parameters of the CNN network.

**Figure 4:**
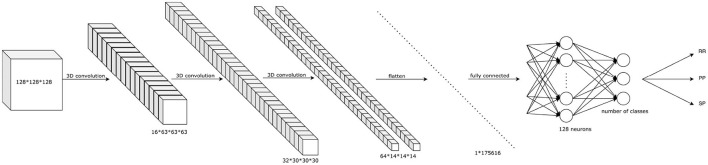
The overall structure of the proposed 3D-CNN network. It starts with three convolutional layers of 16, 32, and 64 filters respectively, each convolution layer followed by a max pooling layer. The tensor is then flattened and two fully connected layers are used to obtain the classification into three classes (RR, PP, SP).

Before using a deep neural network to classify the 3D MRI, all scans were pre-processed using the brain extraction tool (BET) of FMRIB Software Library in order to eliminate non-brain structures. Then, the 3D-CNN image classification network predicts the class (RR, SP, or PP) of the T1w image of a patient's brain used as input. The architecture used is summarized in Figure 4. To prevent over-fitting, a dropout (Srivastava et al., 2014) rate of 0.3 is applied after the flattening layer.

As it is known that CNN classification needs numerous data to perform well, we compared its performance with the classification results using a graph-based neural network.

### 2.4 Experimental settings

According to our previous study using brain morphological connectivity (Barile et al., 2022), 4 threshold levels τ ∈ {0, 0.6, 0.7, 0.8} were applied to the adjacent matrix computed using the 3 atlases and the 2 distances. Thus, each GCN classification is carried out in 72 different ways, and one for CNN.

For both network architectures, the MS images were divided into two datasets: approximately 80% of scans used for training and 20% of the scans used only for testing, i.e., to evaluate the performance of networks. To avoid the impacts of repetition of the same patient, we carefully grouped all time points of one patient in the same train or test set using the stratified group k-fold technique. The exams of the same patient won't be in the train set and test set simultaneously.

The precision, recall, and the F1-score were used to assess both algorithms' effectiveness. To provide a more thorough assessment of the two models, cross-validation using five-folds was performed.

From hyperparameters manual optimization, we use the Adam optimizer with a learning rate of 0.001 for GCN and the Stochastic Gradient Descent optimizer with a learning rate of 0.001 for 3D-CNN.

GCN was trained on one GPU (NVIDIA GeForce RTX 3060), and CNN was trained on one NVIDIA RTX A5000. All experiments were done using PyTorch.

For code availability, the reader can refer to Section 5.

## 3 Results

In this section, we first present the GCN classification tasks and then the results without age and gender normalization to allow the comparison with 3D-CNN classification results. Second, the GCN classification results with age and sex normalization are presented.

### 3.1 Clinical forms classification tasks

Six classification tasks related to clinical needs were implemented: (1) RR vs. PP; (2) RR vs. SP; (3) PP vs. SP; (4) RR vs. PP+SP; (5) RR vs. PP vs. SP; (6) MS vs. HC. For this last task, the train set consists of 619 MS scans and 290 randomly selected scans from the IXI dataset. For the test set, 42 scans were selected from the MS group (24 RRMS, 10 PPMS, 8 SPMS) along with the 21 HC-AMSEP scans from the same study and 24 HC-IXI scans from the IXI dataset. For the other tasks, only the MS patients dataset was used. A five-fold stratified cross-validation scheme was applied for all tasks.

### 3.2 GCN classification

#### 3.2.1 Without normalization

F1-score of the three atlases (Desikan-Killiany, Destrieux, Glasser), four rejection rates and two distance calculation approaches were compared as shown in Tables 3, 4. Precision and Recall measures of corresponding experiments were included in Supplementary material.

**Table 3:** F1-scores (mean value ± standard deviation) of clinical forms classification using GCN based on Mahalanobis graph for three parcellation atlases and four threshold levels τ.

**Atlas**	**Tasks**	**τ = 0**	**τ = 0.6**	**τ = 0.7**	**τ = 0.8**
**Desikan-Killiany**	**RR vs. PP**	**0.701 ± 0.076**	**0.698 ± 0.068**	**0.706 ±0.056**	**0.703 ± 0.052**
	**RR vs. SP**	**0.684 ± 0.064**	**0.7 ±0.077**	**0.684 ± 0.061**	**0.674 ± 0.08**
	**RR vs. PP + SP**	**0.654 ±0.088**	**0.648 ± 0.081**	**0.647 ± 0.081**	**0.638 ± 0.071**
	**RR vs. PP vs. SP**	**0.594 ± 0.047**	**0.593 ± 0.059**	**0.603 ±0.037**	**0.567 ± 0.043**
	**PP vs. SP**	**0.438 ± 0.092**	**0.475 ±0.073**	**0.466 ± 0.064**	**0.465 ± 0.101**
	**MS vs. HC**	**1.000 ± 0.000**	**1.000 ± 0.000**	**1.000 ± 0.000**	**1.000 ± 0.000**
Destrieux	RR vs. PP	0.72 ± 0.103	0.721 ± 0.089	0.721 ± 0.088	**0.725** **±0.085**
	RR vs. SP	0.684 ± 0.065	0.679 ± 0.066	0.666 ± 0.055	**0.686** **±0.07**
	RR vs. PP + SP	0.649 ± 0.074	**0.657** **±0.061**	0.656 ± 0.058	0.642 ± 0.071
	RR vs. PP vs. SP	0.569 ± 0.037	0.588 ± 0.059	0.587 ± 0.057	**0.596** **±0.066**
	PP vs. SP	**0.485** **±0.05**	0.45 ± 0.054	0.479 ± 0.058	0.466 ± 0.073
	MS vs. HC	1.000 ± 0.000	1.000 ± 0.000	1.000 ± 0.000	1.000 ± 0.000
Glasser	RR vs. PP	0.702 ± 0.096	**0.722** **±0.102**	0.711 ± 0.099	0.714 ± 0.079
	RR vs. SP	0.711 ± 0.062	0.71 ± 0.059	0.694 ± 0.071	**0.722** **±0.067**
	RR vs. PP + SP	0.627 ± 0.085	0.681 ± 0.085	0.687 ± 0.084	**0.689** **±0.095**
	RR vs. PP vs. SP	0.609 ± 0.038	0.634 ± 0.055	0.62 ± 0.066	**0.642** **±0.063**
	PP vs. SP	0.495 ± 0.076	0.479 ± 0.076	**0.531** **±0.115**	0.471 ± 0.077
	MS vs. HC	1.000 ± 0.000	1.000 ± 0.000	1.000 ± 0.000	1.000 ± 0.000

The best rejection rates within each atlas are in bold, the best overall results are in gray background.

**Table 4:** F1-scores (mean value ± standard deviation) of clinical forms classification using GCN based on Taxicab graph for three parcellation atlases and four threshold levels τ.

**Atlas**	**Tasks**	**τ = 0**	**τ = 0.6**	**τ = 0.7**	**τ = 0.8**
Desikan-Killiany	RR vs. PP	0.701 ± 0.075	**0.709** **±0.065**	0.706 ± 0.056	0.693 ± 0.097
	RR vs. SP	0.682 ± 0.061	0.671 ± 0.063	**0.684** **±0.061**	0.671 ± 0.052
	RR vs. PP + SP	0.654 ± 0.087	0.662 ± 0.08	**0.667** **±0.073**	0.646 ± 0.078
	RR vs. PP vs. SP	0.596 ± 0.047	0.601 ± 0.04	**0.603** **±0.037**	0.571 ± 0.033
	PP vs. SP	0.437 ± 0.092	0.458 ± 0.07	0.466 ± 0.064	**0.471** **±0.07**
	MS vs. HC	1.000 ± 0.000	1.000 ± 0.000	1.000 ± 0.000	1.000 ± 0.000
Destrieux	RR vs. PP	0.721 ± 0.103	0.719 ± 0.097	**0.721** **±0.088**	0.709 ± 0.075
	RR vs. SP	**0.683** **±0.064**	0.674 ± 0.054	0.666 ± 0.055	0.649 ± 0.064
	RR vs. PP + SP	**0.65** **±0.075**	0.647 ± 0.074	0.649 ± 0.066	0.648 ± 0.064
	RR vs. PP vs. SP	0.569 ± 0.037	**0.587** **±0.055**	0.587 ± 0.057	0.58 ± 0.057
	PP vs. SP	0.481 ± 0.05	0.476 ± 0.057	0.479 ± 0.058	**0.493** **±0.043**
	MS vs. HC	1.000 ± 0.000	1.000 ± 0.000	1.000 ± 0.000	1.000 ± 0.000
Glasser	RR vs. PP	0.701 ± 0.095	**0.722** **±0.096**	0.711 ± 0.099	0.696 ± 0.099
	RR vs. SP	**0.711** **±0.063**	0.708 ± 0.069	0.694 ± 0.071	0.672 ± 0.035
	RR vs. PP + SP	0.628 ± 0.086	**0.656** **±0.09**	0.653 ± 0.096	0.63 ± 0.09
	RR vs. PP vs. SP	0.609 ± 0.039	**0.629** **±0.068**	0.62 ± 0.066	0.593 ± 0.065
	PP vs. SP	0.494 ± 0.073	0.513 ± 0.089	**0.531** **±0.115**	0.526 ± 0.09
	MS vs. HC	1.000 ± 0.000	1.000 ± 0.000	1.000 ± 0.000	1.000 ± 0.000

The best rejection rates within each atlas are in bold, the best overall results are in gray background.

Comparing classification results task by task, the best result was always found using Mahalanobis instead of Taxicab distance for the dissimilarity measurement. The classification of RR vs. PP gave the best result when an 80% rejection rate was applied to the Destrieux atlas with an F1-score of 72.5%. The separation between RR and SP patients provides an F1-score of 72.2% using an 80% rejection rate on the Glasser atlas. By grouping the PP and SP in a neurodegenerative group, the binary classification of RR vs. PP+SP reached an F1-score of 68.9%. The best three classes classification was obtained using an 80% rejection rate on the Glasser atlas with an F1-score of 64.2%. The optimal PP/SP splitting leading to an F1-score of 53.1% was obtained using the Glasser atlas and a rejection rate of 70%. Finally, all GCN classification networks can achieve a great result on MS vs. HC task (100% F1-score on the predefined unseen test dataset). Atlas-wise speaking, for Mahalanobis distance measurement, a 60% rejection rate gave the best result on the Desikan-Killiany atlas, while an 80% rejection rate yielded the best outcome on both Destrieux and Glasser atlases. For Taxicab distance measurement, a 70% rejection rate gave the best result on the Desikan-Killiany atlas, the graph without rejection generated the best on the Destrieux atlas, and a 60% rejection rate achieved the best performance on the Glasser atlas.

#### 3.2.2 With normalization

In order to correct for age and gender, two normalization methods have been carried out. The results obtained using three atlases and two distance methods are shown in Tables 5–8. The best RR/PP separation can be found when the residual normalization was carried out to the Desikan-Killiany atlas with a threshold of 80%. The proportional normalization method applied to the Glasser atlas with an 80% rejection rate generated the best results of RR vs. SP, RR vs. PP+SP, and RR vs. PP vs. SP with F1-scores 71.1, 67.8, and 62.1% respectively. The best result of PP/SP classification can be found in residual normalization on the Desikan-Killiany atlas (rejection rate = 0) with an F1-score of 64.2%. For the proportional normalization method, the best overall result can be found using the Glasser atlas with 80% threshold. The best overall result for the residual normalization method was carried out by the same atlas with 60% threshold.

**Table 5:** F1-scores (mean value ± standard deviation) of clinical forms classification using GCN based on Mahalanobis age-gender proportional adjusted graph for three parcellation atlases and four threshold levels τ.

**Atlas**	**Tasks**	**τ = 0**	**τ = 0.6**	**τ = 0.7**	**τ = 0.8**
Desikan-Killiany	RR vs. PP	0.582 ± 0.091	0.581 ± 0.111	**0.616** **±0.091**	0.611 ± 0.096
	RR vs. SP	**0.613** **±0.08**	0.609 ± 0.07	0.6 ± 0.066	0.591 ± 0.065
	RR vs. PP + SP	0.615 ± 0.058	0.622 ± 0.044	**0.625** **±0.047**	0.592 ± 0.048
	RR vs. PP vs. SP	**0.545** **±0.049**	0.551 ± 0.069	0.535 ± 0.068	0.529 ± 0.049
	PP vs. SP	0.428 ± 0.044	**0.491** **±0.056**	0.45 ± 0.043	0.463 ± 0.083
	MS vs. HC	1.000 ± 0.000	1.000 ± 0.000	1.000 ± 0.000	1.000 ± 0.000
Destrieux	RR vs. PP	0.629 ± 0.118	**0.635** **±0.115**	0.625 ± 0.115	0.605 ± 0.127
	RR vs. SP	0.63 ± 0.076	0.634 ± 0.076	0.632 ± 0.102	**0.647** **±0.105**
	RR vs. PP + SP	**0.608** **±0.068**	0.601 ± 0.05	0.602 ± 0.069	0.589 ± 0.054
	RR vs. PP vs. SP	0.546 ± 0.043	0.548 ± 0.056	0.558 ± 0.061	**0.58** **±0.073**
	PP vs. SP	0.476 ± 0.044	0.471 ± 0.055	**0.494** **±0.058**	0.49 ± 0.066
	MS vs. HC	1.000 ± 0.000	1.000 ± 0.000	1.000 ± 0.000	1.000 ± 0.000
Glasser	RR vs. PP	0.635 ± 0.146	0.668 ± 0.124	0.669 ± 0.122	**0.671** **±0.117**
	RR vs. SP	0.638 ± 0.092	0.679 ± 0.117	0.692 ± 0.114	**0.711** **±0.107**
	RR vs. PP + SP	0.619 ± 0.063	0.643 ± 0.071	0.657 ± 0.075	**0.678** **±0.063**
	RR vs. PP vs. SP	0.578 ± 0.077	0.582 ± 0.065	0.6 ± 0.044	**0.621** **±0.032**
	PP vs. SP	**0.592** **±0.086**	0.569 ± 0.097	0.525 ± 0.09	0.533 ± 0.116
	MS vs. HC	1.000 ± 0.000	1.000 ± 0.000	1.000 ± 0.000	1.000 ± 0.000

The best rejection rates within each atlas are in bold, the best overall results are in gray background.

**Table 6:** F1-scores (mean value ± standard deviation) of clinical forms classification using GCN based on Taxicab age-gender proportional adjusted graph for three parcellation atlases and four threshold levels τ.

**Atlas**	**Tasks**	**τ = 0**	**τ = 0.6**	**τ = 0.7**	**τ = 0.8**
**Desikan-Killiany**	**RR vs. PP**	**0.588 ± 0.089**	**0.581 ± 0.111**	**0.615 ±0.092**	**0.611 ± 0.096**
	**RR vs. SP**	**0.607 ± 0.08**	**0.609 ±0.071**	**0.6 ± 0.066**	**0.591 ± 0.063**
	**RR vs. PP + SP**	**0.615 ± 0.06**	**0.622 ± 0.045**	**0.626 ±0.047**	**0.592 ± 0.047**
	**RR vs. PP vs. SP**	**0.542 ±0.049**	**0.55 ± 0.069**	**0.535 ± 0.068**	**0.529 ± 0.046**
	**PP vs. SP**	**0.427 ± 0.044**	**0.49 ±0.053**	**0.451 ± 0.042**	**0.462 ± 0.083**
	**MS vs. HC**	**1.000 ± 0.000**	**1.000 ± 0.000**	**1.000 ± 0.000**	**1.000 ± 0.000**
Destrieux	RR vs. PP	0.632 ± 0.119	**0.636** **±0.116**	0.631 ± 0.111	0.605 ± 0.128
	RR vs. SP	0.637 ± 0.075	0.633 ± 0.09	0.631 ± 0.101	**0.647** **±0.105**
	RR vs. PP + SP	**0.609** **±0.067**	0.601 ± 0.051	0.601 ± 0.07	0.588 ± 0.054
	RR vs. PP vs. SP	0.546 ± 0.042	0.549 ± 0.056	0.558 ± 0.061	**0.58** **±0.074**
	PP vs. SP	0.48 ± 0.045	0.473 ± 0.057	**0.493** **±0.057**	0.489 ± 0.067
	MS vs. HC	1.000 ± 0.000	1.000 ± 0.000	1.000 ± 0.000	1.000 ± 0.000
Glasser	RR vs. PP	0.618 ± 0.12	0.645 ± 0.098	0.63 ± 0.117	**0.655** **±0.085**
	RR vs. SP	0.627 ± 0.092	0.669 ± 0.11	0.686 ± 0.106	**0.7** **±0.096**
	RR vs. PP + SP	0.606 ± 0.055	0.0.632 ± 0.069	0.649 ± 0.069	**0.67** **±0.059**
	RR vs. PP vs. SP	0.567 ± 0.068	0.572 ± 0.057	0.594 ± 0.039	**0.611** **±0.029**
	PP vs. SP	**0.6** **±0.094**	0.576 ± 0.097	0.538 ± 0.096	0.51 ± 0.101
	MS vs. HC	1.000 ± 0.000	1.000 ± 0.000	1.000 ± 0.000	1.000 ± 0.000

The best rejection rates within each atlas are in bold, the best overall results are in gray background.

**Table 7:** F1-scores (mean value ± standard deviation) of clinical forms classification using GCN based on Mahalanobis age-gender residual adjusted graph for three parcellation atlases and four threshold levels τ.

**Atlas**	**Tasks**	**τ = 0**	**τ = 0.6**	**τ = 0.7**	**τ = 0.8**
**Desikan-Killiany**	**RR vs. PP**	**0.7 ± 0.097**	**0.681 ± 0.097**	**0.679 ± 0.085**	**0.715 ±0.069**
	**RR vs. SP**	**0.578 ± 0.105**	**0.577 ± 0.109**	**0.579 ± 0.114**	**0.581 ±0.126**
	**RR vs. PP + SP**	**0.612 ± 0.055**	**0.618 ±0.064**	**0.603 ± 0.069**	**0.61 ± 0.068**
	**RR vs. PP vs. SP**	**0.525 ±0.065**	**0.484 ± 0.042**	**0.488 ± 0.066**	**0.503 ± 0.055**
	**PP vs. SP**	**0.635 ±0.079**	**0.601 ± 0.09**	**0.595 ± 0.098**	**0.563 ± 0.118**
	**MS vs. HC**	**1.000 ± 0.000**	**1.000 ± 0.000**	**1.000 ± 0.000**	**1.000 ± 0.000**
Destrieux	RR vs. PP	**0.709** **±0.102**	0.693 ± 0.105	0.697 ± 0.107	0.696 ± 0.11
	RR vs. SP	0.58 ± 0.103	0.579 ± 0.11	0.599 ± 0.115	**0.603** **±0.124**
	RR vs. PP + SP	**0.558** **±0.035**	0.557 ± 0.015	0.547 ± 0.008	0.538 ± 0.025
	RR vs. PP vs. SP	0.483 ± 0.074	0.476 ± 0.092	0.481 ± 0.099	**0.49** **±0.101**
	PP vs. SP	0.481 ± 0.105	0.498 ± 0.094	0.505 ± 0.083	**0.528** **±0.077**
	MS vs. HC	1.000 ± 0.000	1.000 ± 0.000	1.000 ± 0.000	1.000 ± 0.000
Glasser	RR vs. PP	**0.711** **±0.087**	0.707 ± 0.098	0.705 ± 0.096	0.644 ± 0.153
	RR vs. SP	0.595 ± 0.132	0.612 ± 0.131	0.619 ± 0.138	**0.637** **±0.127**
	RR vs. PP + SP	0.588 ± 0.08	**0.617** **±0.083**	0.607 ± 0.088	0.608 ± 0.094
	RR vs. PP vs. SP	0.51 ± 0.068	**0.54** **±0.082**	0.537 ± 0.083	0.527 ± 0.066
	PP vs. SP	**0.566** **±0.149**	0.509 ± 0.096	0.523 ± 0.093	0.561 ± 0.097
	MS vs. HC	1.000 ± 0.000	1.000 ± 0.000	1.000 ± 0.000	1.000 ± 0.000

The best rejection rates within each atlas are in bold, the best overall results are in gray background.

**Table 8:** F1-scores (mean value ± standard deviation) of clinical forms classification using GCN based on Taxicab age-gender residual adjusted graph for three parcellation atlases and four threshold levels τ.

**Atlas**	**Tasks**	**τ = 0**	**τ = 0.6**	**τ = 0.7**	**τ = 0.8**
Desikan-Killiany	RR vs. PP	0.7 ± 0.097	0.681 ± 0.097	0.678 ± 0.085	**0.715** **±0.072**
	RR vs. SP	0.579 ± 0.111	0.58 ± 0.106	**0.583** **±0.113**	0.575 ± 0.12
	RR vs. PP + SP	0.611 ± 0.055	**0.617** **±0.062**	0.607 ± 0.067	0.609 ± 0.067
	RR vs. PP vs. SP	**0.525** **±0.065**	0.485 ± 0.042	0.482 ± 0.062	0.503 ± 0.056
	PP vs. SP	**0.642** **±0.079**	0.604 ± 0.088	0.593 ± 0.101	0.567 ± 0.124
	MS vs. HC	1.000 ± 0.000	1.000 ± 0.000	1.000 ± 0.000	1.000 ± 0.000
Destrieux	RR vs. PP	**0.711** **±0.101**	0.693 ± 0.105	0.694 ± 0.112	0.696 ± 0.112
	RR vs. SP	0.582 ± 0.105	0.579 ± 0.112	0.597 ± 0.122	**0.598** **±0.123**
	RR vs. PP + SP	0.553 ± 0.036	**0.56** **±0.015**	0.533 ± 0.025	0.531 ± 0.032
	RR vs. PP vs. SP	**0.491** **±0.073**	0.476 ± 0.092	0.479 ± 0.098	0.48 ± 0.1
	PP vs. SP	0.48 ± 0.106	0.497 ± 0.091	0.526 ± 0.076	**0.527** **±0.074**
	MS vs. HC	1.000 ± 0.000	1.000 ± 0.000	1.000 ± 0.000	1.000 ± 0.000
Glasser	RR vs. PP	**0.713** **±0.088**	0.707 ± 0.098	0.705 ± 0.096	0.645 ± 0.155
	RR vs. SP	0.589 ± 0.126	0.611 ± 0.131	0.618 ± 0.135	**0.637** **±0.128**
	RR vs. PP + SP	0.592 ± 0.086	**0.618** **±0.084**	0.607 ± 0.088	0.608 ± 0.09
	RR vs. PP vs. SP	0.508 ± 0.067	**0.542** **±0.083**	0.537 ± 0.081	0.523 ± 0.062
	PP vs. SP	**0.567** **±0.126**	0.509 ± 0.095	0.529 ± 0.095	0.55 ± 0.088
	MS vs. HC	1.000 ± 0.000	1.000 ± 0.000	1.000 ± 0.000	1.000 ± 0.000

The best rejection rates within each atlas are in bold, the best overall results are in gray background.

### 3.3 Comparing CNN and GCN

The results of the comparison between 3D-CNN classification and GCN without normalization are shown in Table 9. Comparing RR individually with PP and SP, 3D-CNN returned an F1-score of 72.1% and 69.7% respectively, which are slightly lower than GCN results. The separation between the RR and PP+SP groups on the F1-score was greater than that of the GCN technique at 70.7%. The 3D-CNN method generated a similar result on the multi-class classification task with an F1-score of 63.9%. Finally, 3D-CNN achieved a lower result than GCN for the PP vs. SP partition with a 49.5% F1-score. Overall, the best results were obtained using GCN over 3D-CNN while implementing an 80% rejection rate on the Glasser atlas and the Mahalanobis distance.

**Table 9:** Best F1-scores (mean value ± standard deviation) of clinical forms classification using 3D-CNN and GCN [three datasets: non-normalized (NN) graph, proportional normalized (PN) graph, and residual normalized (RN) graph].

**Tasks**	**3D-CNN**	**NN GCN**	**PN GCN**	**RN GCN**
RR vs. PP	0.697 ± 0.124	**0.725** **±0.085**	0.671 ± 0.117	0.715 ± 0.069
RR vs. SP	0.721 ± 0.081	**0.722** **±0.067**	0.711 ± 0.107	0.637 ± 0.128
RR vs. PP + SP	**0.707** **±0.066**	0.689 ± 0.095	0.678 ± 0.063	0.618 ± 0.084
RR vs. PP vs. SP	0.639 ± 0.036	**0.642** **±0.063**	0.621 ± 0.032	0.542 ± 0.083
PP vs. SP	0.495 ± 0.06	0.531 ± 0.115	0.6 ± 0.094	**0.642** **±0.079**

The best F1-scores for each classification task are in bold.

## 4 Discussion

Graph Convolutional Network is an innovative approach for the classification of clinical forms in multiple sclerosis. While functional and structural connectivities were previously used and provided good results (Ktena et al., 2018; Marzullo et al., 2019), they were constrained by the small size of the database available in clinical routine. To overcome this limitation, one approach is to develop a morphological connectivity method requiring only anatomical T1w MRI for brain studies. In order to test such a hypothesis, we developed a complete pipeline using morphological connectivity and graph convolutional networks. To our knowledge, this is the first attempt to use this approach for the classification of MS clinical forms. Brain graphs were established based on Desikan-Killiany, Destrieux, and Glasser atlases, for GM parcellation. Rejection rates of 60, 70, and 80% were applied to connectivity graphs to preserve solely main differences across brain regions. Morphological connectivity data were fed into GCN while 3D brain images were loaded in 3D-CNN to compare the two classification approaches.

First, non-normalized GCN was compared to 3D-CNN, which was unable to normalize age or gender based on image data. Generally speaking, GCN has outperformed 3D-CNN on 4 out of 5 predefined tasks when the threshold/atlas pair was carefully chosen. For the task RR vs. PP+SP, the F1-score generated by GCN was slightly weaker than the result of 3D-CNN with a 1.8 percentage point. However, it requires more computation resources to train a simple 3 convolutional layers network. In our case, GCN only took 5 h for network training while achieving a better result than 3D-CNN which took more than a week on the same computer. The proposed pipeline has gained in computation time thanks to its dimension-reduction ability. Instead of working on 256 × 256 × 256 volumetric images, the graph approach allowed us to use the adjacent matrix of size 360 × 360 in the most complex case.

The comparison of the two classification networks has also given us insights into the medical image processing field. In general, clinical image classification tasks can be easily affected by acquisition changes (manufacturers, centers, MR field, etc.). In particular, CNNs are sensitive to intensity changes with the use of convolution layers. To address this problem, CNN classification networks must be trained on a large number of images that represent both the variability of the acquisition process and the diversity of the patients. Since most medical datasets are composed of a small number of patients, CNN doesn't usually generate well due to its data-thirsty characteristic. In contrast, GCN can be trained on brain graph features that are less sensitive to image intensity changes. Indeed, cortical thinning is an important biomarker of the MS neurodegenerative process that is visible in T1w images (Narayana et al., 2013). With a brain graph generated from cortical thickness, these small changes in the brain were well-captured by the proposed GCN pipeline. Our pipeline returns a clearer relation between brain atrophy and clinical forms, compared to the 3D-CNN approach, which could be improved by using Grad-CAM (Selvaraju et al., 2020) or similar methods.

Second, normalized GCN was used to classify MS clinical forms. This is essential for clinical forms classification. Binary and multi-class classifications were performed between the three clinical forms (RR, PP, SP). The result of normalized GCN showed that GCN can return satisfactory results on binary classification between MS clinical courses. More specifically, the automatic separation of inflammatory forms from neurodegenerative forms, RR vs. SP and PP groups, has been carried out. The best F1-score was found when separating RR from PP patients, and a good result was also obtained in the RR/SP classification task. On one hand, RR patients present relapses corresponding to focal inflammatory processes. On the other hand, SP and PP patients share the experience of progressive clinical evolution, associated or not with inflammatory activity, resulting from degenerative phenomena of the gray matter. Thus, by grouping SP and PP patients, an adequate result was found when the finest atlas (Glasser) was applied.

The three-class classification is a difficult multi-class categorization task which is further worsened by the imbalanced data distribution. Nevertheless, a promising result was obtained using the Glasser parcellation atlas with a high rejection rate, indicating the advantage of dimension reduction when facing complex brain data such as our case.

Classification of SP and PP was the hardest binary classification task to be accomplished. this is partially due to the small amount of PP cases. Indeed, SP and PP are two neurodegenerative forms sharing similar pathological processes. Moreover, PP is a starting clinical form that can be divided into subclasses depending on the level of disability. With an EDSS score ranging from 2 to 7.5, our PP population is composed of both early and late stages of the disease. The latter ones are more relevant and probably more similar to SP patients as shown in the disease duration at scan. This large variability of disability scores reflects different progressions of the disease and thus different stages of brain alterations. Thus, the SP and some PP patients may share MRI phenotypes which makes the classification difficult, and perhaps even unnecessary.

Achieving good results, the binary classification of HC vs. MS patients was not our primary goal. In general, MS patients can be easily distinguished from healthy subjects in both clinical and imaging ways. In our experience, an F1-score of 100% was observed in all GCN outputs, meaning that all combinations of atlases and thresholds provided enough information for the classification task. Similar results were obtained in the previous work of Marzullo et al. (2019) on brain structural connectivity. Marzullo et al. (2019) has performed the test of HC vs. CIS+RR (24/253) and the test of HC vs. SP+PP (24/325) and achieved the best result (F-measure = 1), demonstrating an evident difference between HC and MS brain morphological and structural networks, respectively.

To further compare our work with other studies, we analyzed the results obtained from Marzullo et al. (2019) and Barile et al. (2022). Apart from the binary classification of HC vs. MS patients, Marzullo et al. (2019) have also tested the separation between early and progressive forms of MS (CIS+RR vs. SP+PP: 253/325) obtaining the highest F-measure at 0.99. Since CIS subjects are included in the RR group in our study, we can compare the previous result with our classification task of RR vs. SP+PP (299/361), leading to an F1-score of 0.678. This strong difference in performance demonstrates that white matter inflammation introduced significant information that facilitates the classification of clinical forms in MS. In contrast, the work of Barile et al. (2022) was performed on GM morphological connectivity. Three similar tasks were reported: (1) CIS+RR vs. PP; (2) CIS+RR vs. SP; (3) CIS+RR vs. SP+PP. By employing the same pipeline of graph generation and atlas (Glasser) and an ensemble of machine learning methods, they have obtained an F1-score of 0.661 (0.12), 0.654 (0.12), 0.648 (0.11) for the three tasks, respectively. In our study, we obtained better F1 scores of 0.671 (0.117), 0.711 (0.107), 0.678 (0.063) for the same tasks. This gain in performance (higher F1-score and reduced standard deviation) demonstrated the interest of brain graph convolutional networks.

Taxicab distance is an L1-norm metric that is generally preferred over Euclidean distance for high-dimension data analysis (Aggarwal et al., 2001). However, since every dimension (mean, standard deviation, skewness, kurtosis) has the same attribution in the calculation of Taxicab distance, our feature vector of four dimensions could not have the same impact on the final value due to the difference in magnitude. In such cases, Mahalanobis distance can overcome the problem while removing redundant information from correlated variables. Since distance measurement was included as edge weight in the input data of GCN, the choice can surely affect the final result. Thus, it is not surprising to observe a better result with Mahalanobis distance supporting the graph generation.

Finally, this work presents several methodological limitations. First the classification results were biased by the class imbalance of the database and the insufficient number of patients. Since the current database consists of a series of multiple MR scans per patient, it does not cover enough variability of the disease, meaning a lack of global vision of the disease. Hence, even if we carefully stop the network training before overfitting, it is hard to extract sufficient features of each MS clinical course to classify an unseen patient by the proposed network, resulting in bad output in some cases. Nevertheless, our cohort study had no bias related to the protocol acquisition, which is unique, guaranteeing the homogeneity of the data. In contrast, a multi-center study is more variable and therefore requires a precise study and corrections of bias.

## 5 Conclusion

Although studies on MS mainly focus on white matter and lesion analysis, morphological change in gray matter is a non-negligible aspect of the disease. A full pipeline was proposed in this study for the classification of MS clinical forms. It starts from automatic GM segmentation and surface parcellation, followed by GM thickness analysis using three different granularity of atlases, two different distance measurements, and two different age-gender normalization methods. Thus, a brain resulted in a morphological connectivity graph accompanied by a feature matrix per graph. Four rejection rates corresponding to noise elimination were applied to the graph. A graph convolutional network was performed on these graphs to exploit the hidden information behind GM morphological features. In parallel, a classic 3D convolutional neural network was applied to the brain MRI directly for comparison. The best results were generated by proportional GCN that trained on Glasser parcellation-based graphs with Mahalanobis distance measurement and 80% rejection rate. In future studies, to fully exploit its capacity for clinical image analysis, our method can be implemented on a larger database to predict patients' disease evolution and obtain the correlation between images' information and patients' disability. However, to work with such a heterogeneous study will require developing more advanced graph networks (i.e., with attention) to limit biases such as gender, age and acquisition systems.

## Data availability statement

The sources code and graph data supporting the conclusions of this article can be found at: https://gitlab.in2p3.fr/thomas.grenier/msgcn-classification.

## Ethics statement

The studies involving humans were approved by Local Ethics Committee (CPP Sud-Est IV) and French National Agency for Medicine and Health Products Safety (ANSM). The studies were conducted in accordance with the local legislation and institutional requirements. The participants provided their written informed consent to participate in this study.

## Author contributions

EC: Conceptualization, Investigation, Software, Writing—original draft, Data curation, Formal analysis, Methodology, Validation, Visualization, Writing—review & editing. BB: Software, Writing—review & editing, Data curation. FD-D: Funding acquisition, Supervision, Writing—review & editing, Validation, Visualization. TG: Methodology, Supervision, Validation, Writing—review & editing, Conceptualization, Formal analysis, Investigation, Visualization. DS-M: Conceptualization, Funding acquisition, Methodology, Project administration, Supervision, Validation, Writing—review & editing, Resources.
