# Supplementary material for: Multiple sclerosis clinical forms classification with graph convolutional networks based on brain morphological connectivity
Source: Front Neurosci. 2024 Jan 18;17:1268860. doi: 10.3389/fnins.2023.1268860 (PMC10830765; doi:10.3389/fnins.2023.1268860)
Supplement: Supplementary file 1 [file Data_Sheet_1.PDF]

Supplementary Material

1 SUPPLEMENTARY TABLES AND FIGURES

1.1 Tables

Atlas	Tasks	$\tau = 0$	$\tau = 0.6$	$\tau = 0.7$	$\tau = 0.8$
Desikan-Killiany	RR vs. PP	0.722±0.083	0.721±0.086	0.723±0.089	0.737±0.059
	RR vs. SP	0.716±0.068	0.733±0.081	0.731±0.085	0.710±0.081
	RR vs. PP + SP	0.678±0.086	0.676±0.080	0.673±0.077	0.664±0.068
	RR vs. PP vs. SP	0.547±0.033	0.558±0.040	0.545±0.028	0.529±0.025
	PP vs. SP	0.402±0.113	0.463±0.092	0.432±0.101	0.481±0.104
	MS vs. HC	1.000±0.000	1.000±0.000	1.000±0.000	1.000±0.000
Destrieux	RR vs. PP	0.753±0.111	0.744±0.102	0.74±0.104	0.748±0.099
	RR vs. SP	0.726±0.074	0.705±0.077	0.707±0.084	0.716±0.082
	RR vs. PP + SP	0.681±0.064	0.684±0.053	0.681±0.051	0.670±0.066
	RR vs. PP vs. SP	0.529±0.032	0.543±0.043	0.55±0.045	0.551±0.049
	PP vs. SP	0.511±0.063	0.425±0.075	0.45±0.087	0.450±0.075
	MS vs. HC	1.000±0.000	1.000±0.000	1.000±0.000	1.000±0.000
Glasser	RR vs. PP	0.717±0.105	0.744±0.121	0.734±0.110	0.731±0.091
	RR vs. SP	0.752±0.056	0.740±0.066	0.736±0.072	0.742±0.071
	RR vs. PP + SP	0.657±0.079	0.704±0.083	0.707±0.084	0.706±0.095
	RR vs. PP vs. SP	0.564±0.026	0.581±0.039	0.587±0.051	0.589±0.051
	PP vs. SP	0.463±0.079	0.447±0.073	0.391±0.069	0.405±0.079
	MS vs. HC	1.000±0.000	1.000±0.000	1.000±0.000	1.000±0.000

Table S1. Precision (mean value ± standard deviation) of clinical forms classification using GCN based on Mahalanobis graph for three parcellation atlases and four threshold levels τ.

Atlas	Tasks	$\tau = 0$	$\tau = 0.6$	$\tau = 0.7$	$\tau = 0.8$
Desikan-Killiany	RR vs. PP	0.721±0.067	0.718±0.065	0.721±0.065	0.731±0.044
	RR vs. SP	0.696±0.061	0.712±0.075	0.710±0.078	0.689±0.076
	RR vs. PP + SP	0.664±0.083	0.660±0.073	0.658±0.075	0.648±0.068
	RR vs. PP vs. SP	0.682±0.061	0.673±0.068	0.669±0.060	0.654±0.055
	PP vs. SP	0.516±0.102	0.544±0.063	0.541±0.071	0.546±0.107
	MS vs. HC	1.000±0.000	1.000±0.000	1.000±0.000	1.000±0.000
Destrieux	RR vs. PP	0.738±0.108	0.737±0.092	0.735±0.095	0.742±0.090
	RR vs. SP	0.699±0.065	0.692±0.062	0.695±0.070	0.698±0.071
	RR vs. PP + SP	0.658±0.074	0.666±0.059	0.664±0.056	0.651±0.067
	RR vs. PP vs. SP	0.651±0.036	0.673±0.073	0.683±0.076	0.685±0.081
	PP vs. SP	0.570±0.055	0.537±0.057	0.554±0.060	0.558±0.063
	MS vs. HC	1.000±0.000	1.000±0.000	1.000±0.000	1.000±0.000
Glasser	RR vs. PP	0.724±0.091	0.742±0.099	0.733±0.086	0.735±0.072
	RR vs. SP	0.724±0.058	0.719±0.061	0.720±0.065	0.728±0.067
	RR vs. PP + SP	0.636±0.085	0.684±0.084	0.692±0.083	0.693±0.095
	RR vs. PP vs. SP	0.703±0.048	0.730±0.063	0.734±0.076	0.739±0.073
	PP vs. SP	0.608±0.071	0.588±0.072	0.592±0.063	0.598±0.060
	MS vs. HC	1.000±0.000	1.000±0.000	1.000±0.000	1.000±0.000

Table S2. Recall (mean value ± standard deviation) of clinical forms classification using GCN based on Mahalanobis graph for three parcellation atlases and four threshold levels τ.

Atlas	Tasks	$\tau = 0$	$\tau = 0.6$	$\tau = 0.7$	$\tau = 0.8$
Desikan-Killiany	RR vs. PP	0.722±0.082	0.731±0.078	0.722±0.066	0.710±0.107
	RR vs. SP	0.716±0.069	0.709±0.070	0.72±0.067	0.702±0.057
	RR vs. PP + SP	0.678±0.086	0.682±0.080	0.691±0.068	0.672±0.072
	RR vs. PP vs. SP	0.547±0.034	0.559±0.024	0.564±0.022	0.533±0.025
	PP vs. SP	0.402±0.112	0.412±0.099	0.448±0.078	0.468±0.079
	MS vs. HC	1.000±0.000	1.000±0.000	1.000±0.000	1.000±0.000
Destrieux	RR vs. PP	0.753±0.110	0.743±0.108	0.742±0.097	0.725±0.086
	RR vs. SP	0.725±0.074	0.705±0.069	0.697±0.067	0.685±0.079
	RR vs. PP + SP	0.682±0.064	0.674±0.063	0.674±0.056	0.676±0.052
	RR vs. PP vs. SP	0.529±0.033	0.542±0.043	0.544±0.048	0.539±0.048
	PP vs. SP	0.512±0.063	0.475±0.050	0.532±0.073	0.551±0.049
	MS vs. HC	1.000±0.000	1.000±0.000	1.000±0.000	1.000±0.000
Glasser	RR vs. PP	0.716±0.105	0.745±0.112	0.735±0.114	0.718±0.110
	RR vs. SP	0.752±0.057	0.735±0.073	0.723±0.074	0.706±0.044
	RR vs. PP + SP	0.656±0.079	0.681±0.087	0.674±0.096	0.656±0.090
	RR vs. PP vs. SP	0.564±0.025	0.575±0.051	0.569±0.051	0.547±0.055
	PP vs. SP	0.463±0.101	0.519±0.131	0.517±0.145	0.533±0.085
	MS vs. HC	1.000±0.000	1.000±0.000	1.000±0.000	1.000±0.000

Table S3. Precision (mean value ± standard deviation) of clinical forms classification using GCN based on Taxicab graph for three parcellation atlases and four threshold levels τ.

Atlas	Tasks	$\tau = 0$	$\tau = 0.6$	$\tau = 0.7$	$\tau = 0.8$
Desikan-Killiany	RR vs. PP	0.722±0.065	0.729±0.057	0.720±0.065	0.718±0.086
	RR vs. SP	0.693±0.059	0.684±0.063	0.710±0.076	0.683±0.051
	RR vs. PP + SP	0.663±0.082	0.670±0.074	0.658±0.075	0.655±0.074
	RR vs. PP vs. SP	0.685±0.060	0.688±0.042	0.668±0.060	0.653±0.043
	PP vs. SP	0.517±0.105	0.549±0.048	0.542±0.071	0.546±0.033
	MS vs. HC	1.000±0.000	1.000±0.000	1.000±0.000	1.000±0.000
Destrieux	RR vs. PP	0.738±0.108	0.735±0.097	0.734±0.093	0.725±0.076
	RR vs. SP	0.698±0.065	0.689±0.052	0.694±0.070	0.664±0.067
	RR vs. PP + SP	0.659±0.075	0.657±0.068	0.664±0.055	0.658±0.057
	RR vs. PP vs. SP	0.651±0.035	0.672±0.064	0.683±0.075	0.664±0.062
	PP vs. SP	0.568±0.052	0.562±0.041	0.553±0.060	0.559±0.047
	MS vs. HC	1.000±0.000	1.000±0.000	1.000±0.000	1.000±0.000
Glasser	RR vs. PP	0.722±0.091	0.742±0.093	0.734±0.086	0.721±0.093
	RR vs. SP	0.725±0.059	0.718±0.070	0.721±0.066	0.685±0.036
	RR vs. PP + SP	0.637±0.086	0.664±0.088	0.692±0.082	0.639±0.090
	RR vs. PP vs. SP	0.704±0.048	0.726±0.075	0.734±0.075	0.681±0.070
	PP vs. SP	0.607±0.071	0.609±0.071	0.591±0.063	0.600±0.067
	MS vs. HC	1.000±0.000	1.000±0.000	1.000±0.000	1.000±0.000

Table S4. Recall (mean value ± standard deviation) of clinical forms classification using GCN based on Taxicab graph for three parcellation atlases and four threshold levels τ.

1.2 Figures

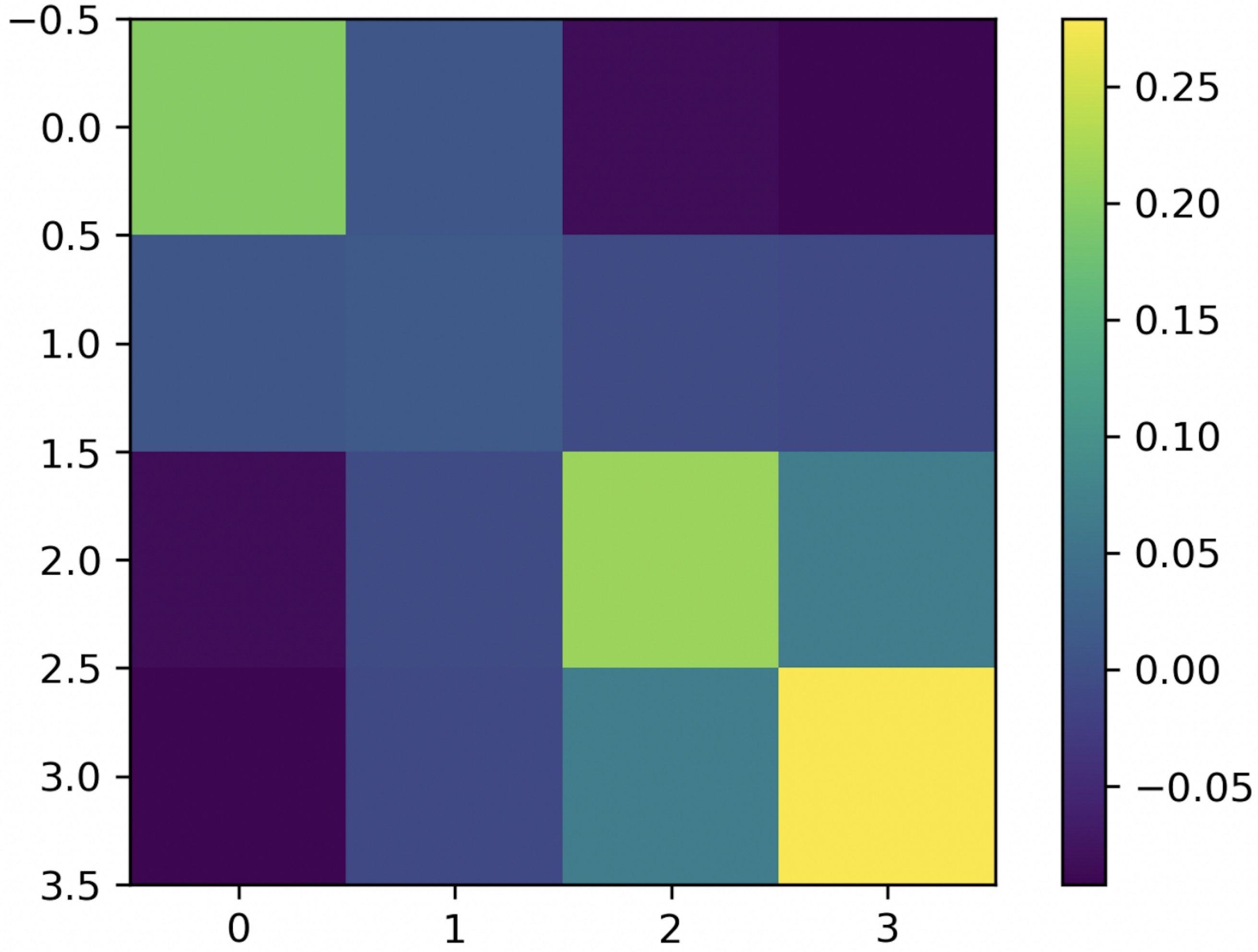

Figure S1: An example of the covariance matrix for the calculation of Mahalanobis distance of a healthy subject from the healthy control group.

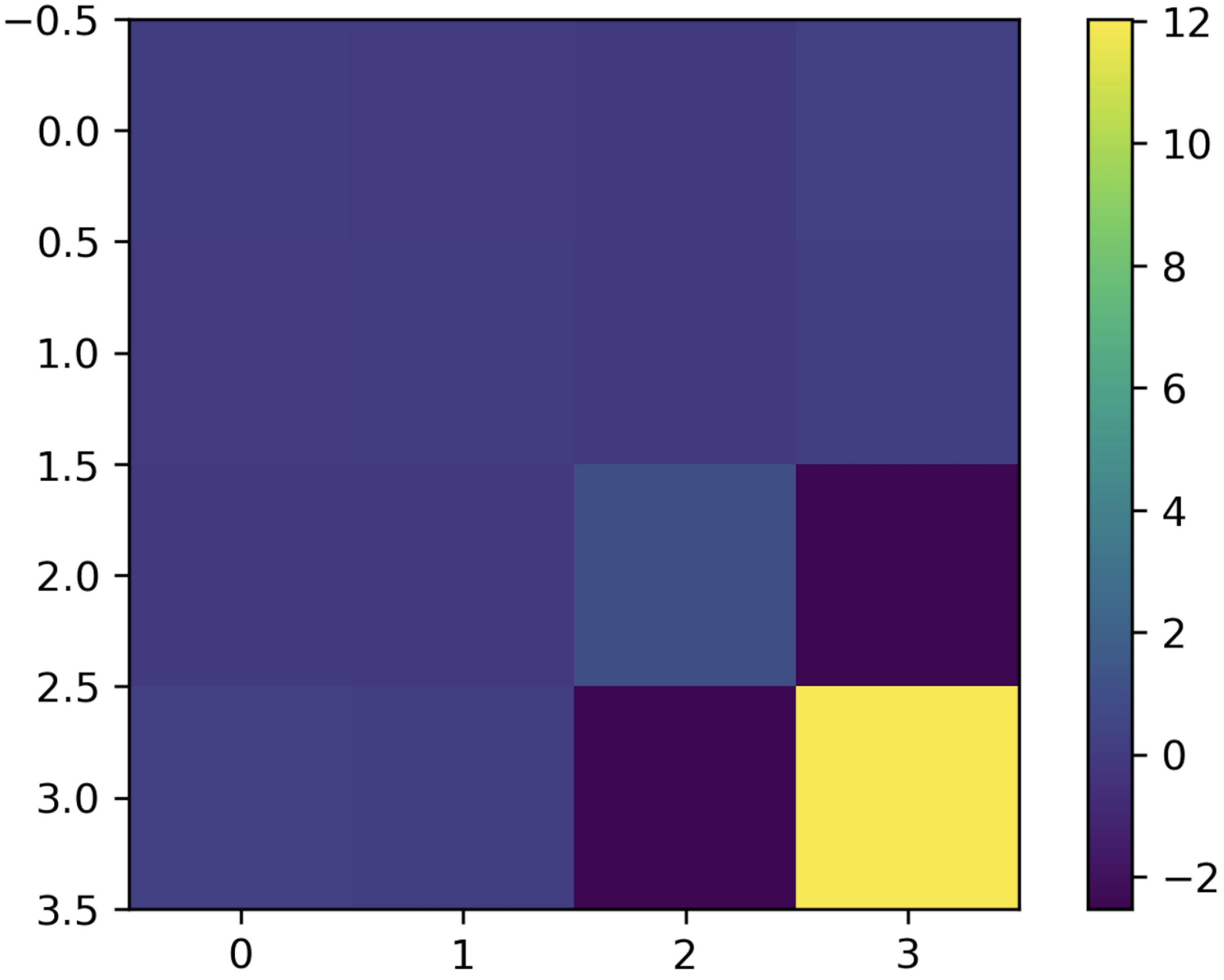

Figure S2: An example of the covariance matrix for the calculation of Mahalanobis distance of an SPMS patient from the MS group.
